# Effect of restoration technique on resistance to fracture of endodontically treated anterior teeth with flared root canals

**DOI:** 10.7555/JBR.32.20170099

**Published:** 2019

**Authors:** Sary S Borzangy, Samah M Saker, Walid A Al-Zordk

**Affiliations:** 1. Department of Substitutive Dental Sciences, College of Dentistry, Taibah University, Medina 42353, Saudi Arabia; 2. Department of Fixed Prosthodontics, Faculty of Dentistry, Mansoura University, Mansoura 35516, Egypt.

**Keywords:** anatomical post, customized post, endodontically treated teeth, flared root canal

## Abstract

This study was designed to compare the impact of post and core systems on resistance to fracture of endodontically treated anterior teeth with flared root canals and to assess their fracture pattern. Sixty central incisors were cut horizontally 2 mm coronal to the cementoenamel junction (CEJ). After root canal therapy, teeth were assigned into 6 groups (*n*=10 each) based on a post system and used as follows: Group C, non-flared root received size #1 glass fiber posts (Control); Group AP, flared root restored with anatomical post; Group RC, flared root restored with size #1 fiber post and cemented with thick layer of resin cement; Group CR, flared root restored with size #1 and reinforced with composite resin; Group CM, cast post-core; Group CP, CAD/CAM polymer-infiltrated ceramic post and core. Following post cementation, core build-up and crown insertion, the specimens were thermo-cycled up to 10,000 cycles (5C/55C; 30 seconds dwell time, 6 seconds transition time) and then statically loaded at 1 mm/minute crosshead speed using a universal testing machine. One-way ANOVA and Tukey HSD post hoc test (α=0.05) were used for data analysis. Group C recorded significantly higher resistance to fracture values [(826.9±39.1) N] followed by group CP [(793.8±55.6) N] while group RC yielded the lowest fracture resistance values [(586.7±51.4) N]. The resistance to fracture of wide root canals can be enhanced by using one-piece CAM/CAM post and core as an alternative to the use of either glass fiber post, relined with composite resin increasing the thickness of luting cement or the use of cast post and core system. However, this was an* in vitro* investigation and further *in vivo* studies are necessary.

## Introduction

Long-term clinical performance of root-canal treated teeth depend primarily on the amount of the tooth’s remaining structure. Teeth with extensive tooth loss have reduced capacity to resist forces during function and a post is essential to hold an artificial core that will restore the tooth loss^[1^–^5]^. For many years, many dental practitioners believed that a post would “reinforce” the tooth structure^[6^–^7]^, however, the volume of dentin remaining is of most relevance to tooth strength^[8^–^11]^. During post space preparation procedure, it is recommended that the remaining dentin should be preserved as much as possible. Resistance to fracture of root canal-filled teeth depends primarily on the remaining root dentin thickness, especially in the bucco-lingual direction^[12]^. The cervical portion of the root canal may be left widely prepared and surrounded only by a thin dentinal wall. And as a result of over-preparation of root canal, removal of extensive caries, and recurrent caries around the post into the root canal dentin thus, reduce the fracture resistance^[2^,^9^,^13^–^14]^. Vertical root fracture of endodontically treated teeth, with wide root canals, represents a critical clinical dilemma^[15]^, although the use of fiber posts (FP) has improved the long-term clinical success^[8]^.

Many studies have been conducted in order to develop a reliable technique of reinforcing endodontically treated teeth with wide root canals^[16^–^19]^. Grandini *et al.*^[20]^ described a technique for reconstruction of compromised endodontically treated tooth by using an “anatomical post”, where a composite resin was used for FP relining so the shape of the post-core matched those of a flared post space. This technique is undoubtedly effective to minimize the volume of luting cement, and reduce its polymerization shrinkage^[21^–^29]^.

In addition, various materials have been recommended in order to fill flared root canal with a biocompatible material with similar physical properties to those of dentin to increase the resistance fracture of weakened roots like composite resins and glass ionomer cements^[19^,^27^,^29]^. The post system chosen to retain a restoration must present biomechanical properties similar to dentin and provide adequate retention and stiffness to prevent any micro-movement between an artificial crown and a root face. Glass fiber post systems were introduced with advantages in respect to their biomechanical properties and to transmit light similar to that of tooth structure^[6^–^9]^.

Recently, Chen *et al.*^[30]^, studied a novel technique to fabricate a custom glass fiber post-and-core with CAD/CAM system for restoration of compromised endodontically treated root canal *in vivo* and they reported the effectiveness of this technique to be used for restoration of flared root canal-treated teeth.

Therefore, this investigation was conducted to evaluate fracture resistance of anterior teeth with flared root canals restored with glass fiber posts, cast post and core and CAD/CAM post and core fabricated from VITA Enamic. The null hypothesis tested was there would be no significant difference in fracture resistance between the four different materials used. Fracture pattern were also evaluated for each test group.

## Materials and methods

### Specimens preparation

Sixty extracted intact human maxillary central incisors of similar-sized, devoid of caries or cracks, with straight root canal were used in this study. The teeth were debrided, cleaned with ultrasonic scaler and brush and residual tissue tags were scraped from the roots. After cleaning, the teeth were stored in a 0.1% thymol solution at 4 °C up to 3 months following extraction before use. Coronal portions of the teeth were transversely cut 2 mm incisal to the cementoenamel junction (CEJ) with a low-speed, water-cooled diamond disk followed by flattening of the sectioned surfaces with silicon carbide paper (600 grit).

### Root canal obturation

After pulpal tissue removal, a step-back technique was used for root canal preparation using final file size number 50 (Dentsply Maillefer, Ballaigues, Switzerland) at the working length that was set at 1 mm away from the apical foramen. A 5.25% NaOCI irrigant was used after each file and up to the final size, followed by rinsing with distilled water. The prepared root canals were obturated with gutta-percha cones (Dentsply Maillefer, Ballaigues, Switzerland) after completely drying with paper points using a lateral condensation technique and AH-Plus sealer (Dentsply IH Ltd, United Kingdom). After obturation, the coronal opening of the root canal was closed with a temporary restorative material (Cavit-G, 3M ESPE, Minnesota, United States) and the teeth were stored for 7 days at 37 °C and at 100% humidity.

### Study groups

**Fig.1:**
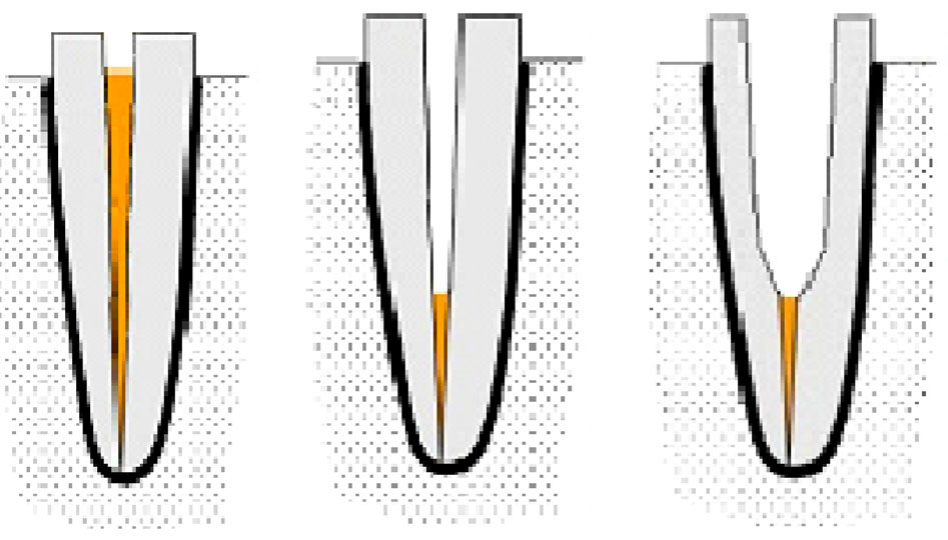
Schematic diagram showing the steps of root canal obturation, gutta-percha removal and flaring of the post space.

To simulate the periodontal ligaments effect, a thin coat of polyvinylsiloxane impression material was painted on the root surface. With the aid of a centralization device, the specimens were embedded vertically in an epoxy resin (Kemapoxy, CMB chemicals, Giza, Egypt) at a depth of 2 mm apical the CEJ. To standardize the post space preparation, the endodontically treated canal were initially prepared to receive a size #1 post (RelyX^TM^, 3M ESPE, St. Paul, Minnesota, United State) of 1.3 mm diameter using matching stainless steel low-speed reamers and the depth was limited to 10 mm using a rubber stopper as a reference (***Fig. 1***). The preparation drill was changed after five preparations so each group used two drills. The specimens were assigned into 6 groups (*n* = 10). Group C: control, non-flared roots; groups AP, RC, CR, CM and CP: flared roots in which the roots were further prepared to simulate roots with flared post space using 2.5 mm diamond burs (#413, KG Sorensen, São Paulo, SP, Brazil) in a low speed hand piece limited to 10 mm vertical length to achieve approximately 1 mm wide coronal root dentinal wall (***Fig. 1***).

### Restoration of the prepared post space

Before post insertion, the posts were airborne-particle abraded with 50-µm alumina particles (Heraeus Kulzer) using a custom-made revolving wheel at 250 kPa pressure for 5 seconds.

Group C (Control, *n*=10): the specimens of this group were restored with size #1 fiber posts (RelyX^TM^, 3M ESPE, St. Paul, Minnesota, United State).

**Fig.2:**
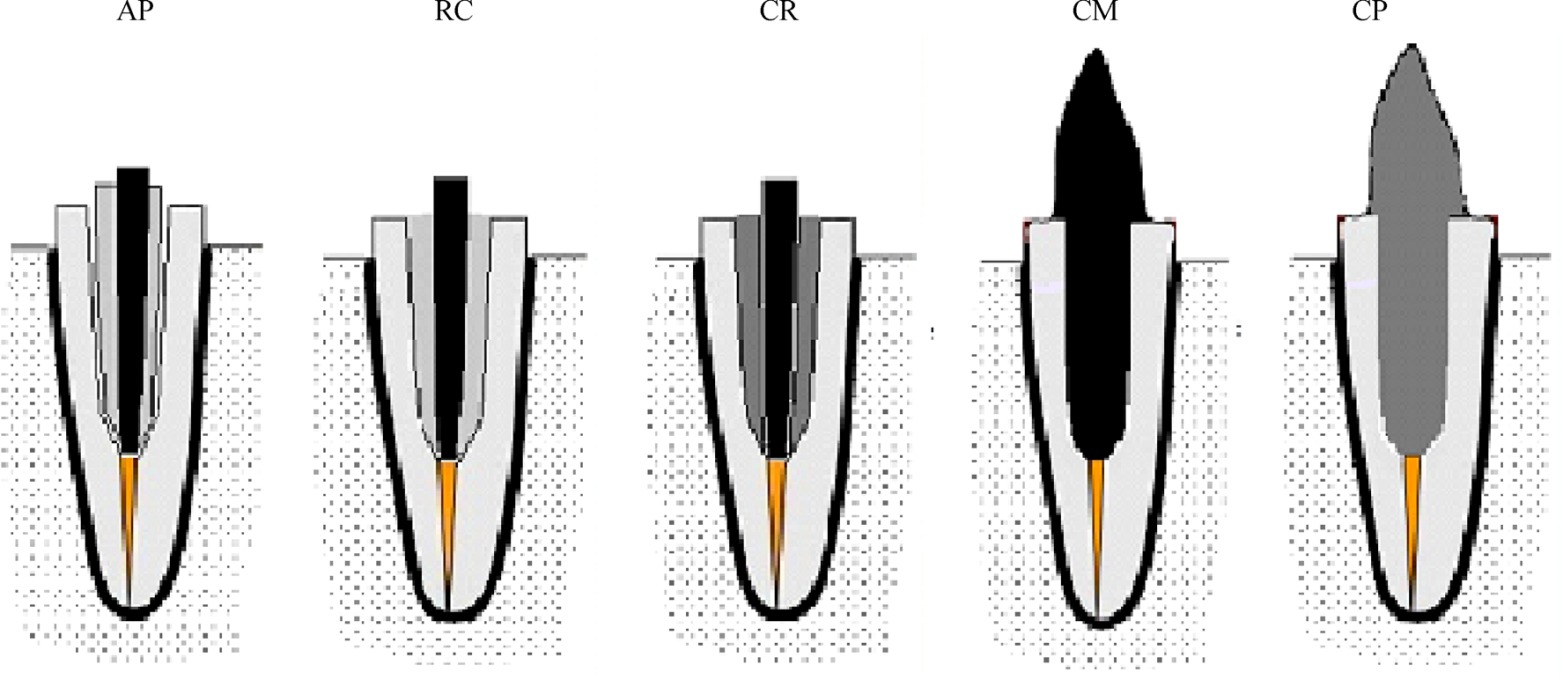
Schematic illustration of the flared roots restored with: anatomical post (AP); size #1 fiber post cemented with thick layer of resin cement (RC); size #1 fiber post reinforced with composite resin (CR); cast post and core (CM); CAD/CAM polymer-infiltrated ceramic post and core.

Group AP (*n*=10): flared post spaces were restored using size #1 fiber posts, relined with composite resin. The fiber post was coated with a layer of silane coupling agent (Prosil, FGM) for 1 minute, and it was gently air dried for 5 seconds and coated with two-step etch-and-rinse bonding agent (Tetric N-Bond, Ivoclar Vivadent), followed by solvent evaporation and finally light cured for 10 seconds. The fiber posts were covered with composite resin (Tetric N-Ceram, Ivoclar Vivadent) then inserted into the canal that previously lubricated with a water-soluble gel (KY, Johnson & Johnson). A customized centralization device was used to aid in guiding and positioning of the lined post in the center of the prepared post space with an even thickness of the composite resin around the post. To insure accurate fitness, the relined fiber post was removed and replaced twice. After removal of the excess resin composite material, the relined fiber post was light cured for 20 sec with the post inside the post space followed by additionally curing for 20 seconds from the buccal, lingual, mesial, and distal surfaces outside the post space. The root canals and the relined fiber posts were then rinsed abundantly with water to remove the lubricant gel (***Fig. 2***).

Group RC (*n* = 10): flared post spaces were restored using size #1 fiber posts cemented with self-adhesive dual-polymerizing resin luting material RelyX Unicem (3M ESPE, Seefled, Germany). The cement capsule was activated and mixed for 15 seconds according to manufacturer’s instructions. The mixed cement was injected into the prepared post space through the elongation tip of the capsule to ensure void-free cementation of the posts (***Fig. 2***). The centralization device was used for positioning of the post within the center of the post space. After removal of excess cement, final light-polymerization was done for 20 seconds on each surface as mentioned previously.

Group CR: reinforcement of flared root canal with composite resin. The dentin walls of the post space were etched for 15 seconds with 37% phosphoric acid gel (Total Etch, Ivoclar Vivadent; Schaan, Liechtenstein) then rinsed and dried with paper points. The two-step etch-and-rinse bonding material was applied on the entire canal surface in two coats with the aid of a microbrush, as recommended by the manufacturer. After solvent evaporation, the adhesive was light cured for 10 seconds by positioning the tip of the light-curing unit at the entrance of the root canal. A flowable composite resin (Tetric N-Flow, Ivoclar Vivadent) was used to fill in the post space from the apex to the cervical portion to avoid the formation of air voids. After this, the glass-fiber post #1 was lubricated with the water-soluble gel (KY, Johnson & Johnson) and inserted into the canal by using a centralization device. The flowable composite resin was light cured for 20 seconds after the removal of the fiber post, the root canals were rinsed abundantly with water to remove the lubricant gel. The fiber posts were rinsed and cleaned. Afterwards, the post spaces were slightly re-prepared with size #0 drill corresponding to the #0.5 post.

Group CM: the canals were lubricated and the autopolymerizing resin (DuraLay II, Reliance Dental Mfg Co) was added over the plastic dowel using bead-brush technique. The pattern was removed inspected after 3 minutes according to instructions of the manufacturer. After refitting the post, the core portion was formed by addition of more pattern resin. The final shaping of the core was done with finishing burs. The patterns were invested and casted with Ni-Cr alloy (Kera NH; Eisenbacher Dentalwaren).

Group CP: the patterns of posts and cores were fabricated as described in group CM. The patterns were sprayed with anti-reflection scan powder spray (Digiscan-Spray, Yeti). Each post-core was mounted over the scanning tray of the extraoral scanner (Ceramill map400, Amann Girrbach). The scanning process was performed automatically by moving the shifting plate with the pattern fixed on it and gaining multiple camera screening. The construction data to design the restorations was performed through standard transformation language (STL) data format. The job definition on the CAD/CAM software (Ceramill Mind software, Amann Girrbach) was started by selection of inlay/onlay as restoration type. After the software calculated the proposed design of post and core, some modifications were made manually. The STL file was sent to the milling unit (Ceramill Motion 2 5X, Amann Girrbach) to mill the post and core from VITA Enamic block. After finishing, the post surfaces were conditioned for 60 seconds with hydrofluoric acid (IPS Ceramic Etching gel, Ivoclar Vivadent) and subsequent silainization (vitigue silane primer, DMG Hamburg, Germany) was done (***Fig. 2***).

Self-adhesive resin cement was used for posts cementation following the instructions of the manufacturer. The composite resin cores (Filtek Z250; 3M ESPE) were fabricated in a standard manner for all groups except groups CM and CP using core-forming matrices (Ultra-form; Ultradent Products Inc). All specimens were prepared to receive complete cast crowns preparation with 0.5 mm width using a diamond bur at high speed with water spray.

### Crown fabrication and cementation

Wax pattern of each crown was made directly on the specimen with a polyvinyl siloxane putty index (Lab Putty; Coltène/Whaledent), to simulate the anatomy of central incisor with an 11 mm length, and a lingual notch 3 mm apical to the incisal edge. To standardize the dimensions, each wax pattern was measured with a wax caliper. The wax patterns were invested and cast in Ni-Cr alloy following the manufacturer’s instructions. The inner surfaces cast crowns were airborne-particle abraded at 0.25 MPa pressure with 150 mm alumina particles (Al2O3; Heraeus Kulzer) and ultrasonically cleaned in 96% isopropanol. The prepared tooth surfaces were cleaned with pumice, rinsed, and dried before final cementation with resin cement following the manufacturer’s recommendations. The specimens were thermocycled up to 10,000 cycles (5C/55C; 30 seconds dwell time, 6 seconds transition time).

### Fracture strength test

**Fig.3:**
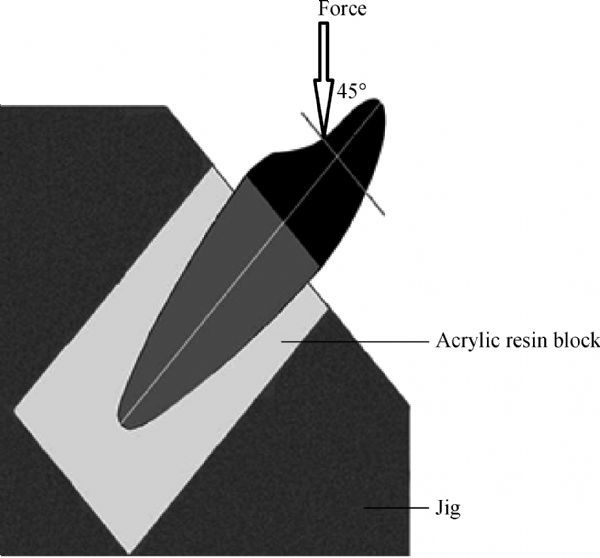
Schematic illustration of the fracture resistance test showing the load applied at 135-degree from the long axis of the root and 3 mm away from the incisal edge.

A standard testing machine (Lloyd Instrument, LTD, West Fareham, UK) was used at a crosshead speed of 1.0 mm/minute with a 5 kN load cell to apply a unidirectional static load at an angle of 135 degrees from the long axis of the root (***Fig. 3***), applied to a groove located at the palatal concavity of the crown located 3.0 mm away from the incisal edge of the restoration. The load was applied until fracture. The force at which initial root fracture occurred was recorded in Newtons (N). Specimens were visually inspected to determine the location, type, and direction of failure. The failure mode was recorded and classified as either “repairable or favorable” (detachment of the post and core and/or fracture of cervical third of the root that could be repaired through fabrication of a new restoration) or “irreparable or catastrophic” (vertical or horizontal fracture and oblique fracture below the cervical third of root that would necessitate extraction of the remaining tooth structure).

### Statistical analysis

The homogeneity of data were evaluated using Kolmogorov-Smirnov and Levene normality tests. Statistical analysis was performed with software (SPSS 20.0, SPSS Inc, Chicago, IL). Fracture resistance data (N) were analyzed by one-way analysis of variance (ANOVA) followed by the Tukey HSD post hoc test (α=0.05) multiple comparisons.

## Results

Means and standard deviations of resistance to fracture values recorded for the test groups were listed in (***Table 1***). Kolmogorov-Smirnov and Levene normality tests revealed normal and relative (marginal) distributions of data. Therefore, a parametric ANOVA analysis test was conducted to evaluate the difference of fracture resistance values among groups (***Table 2***).

**Tab.1:** Mean and standard deviations of fracture resistance values (N) of experimental groups

Groups	N	Mean
Control	10	826.9±39.1^a^
Anatomical post	10	686.2±52.5^b^
Resin cement	10	607.7±54.8^c^
Composite resin	10	727.4±64.3^b^
Cast post and core	10	771.±77.3^d^
CAD/CAM post and core	10	793.8±55.6 ^a^

Significant difference (*P*≤0.05) was found among groups marked by different letters (a, b, c) while no significant difference was found among groups marked by similar letters.

**Tab.2:** Percentage of failure modes observed within each experimental group

Group	Percentage of failure modes (%)	
	Favorable	Non-favorable
Control	80	20
Relined post	60	40
Composite resin	70	30
Resin cement	40	60
Cast post and core	30	70
CAD/ CAM post and core	70	30

**Fig.4:**
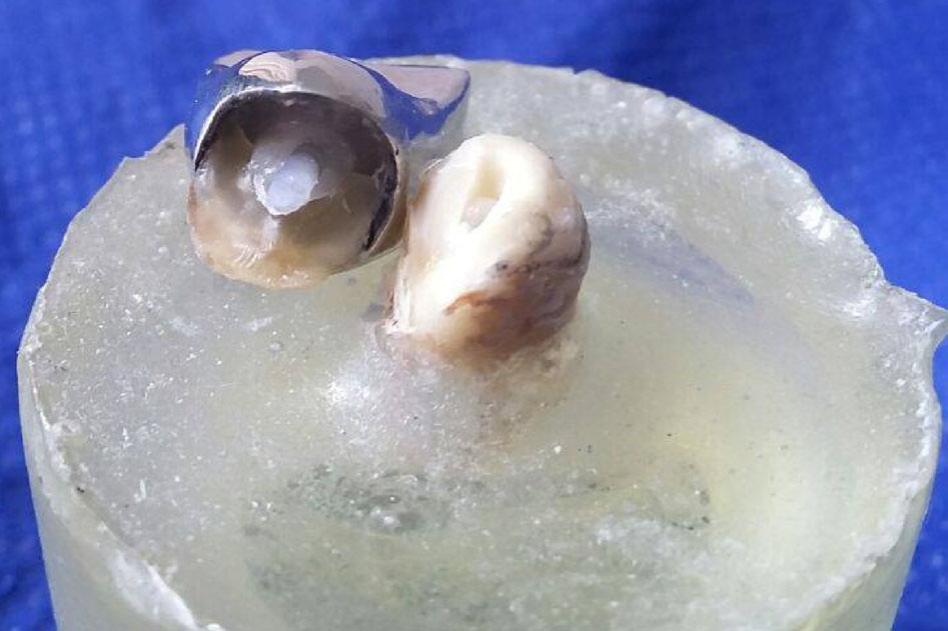
Photograph representing an example of a favorable type of failure with detachment of the post and core system that could be repaired.

**Fig.5:**
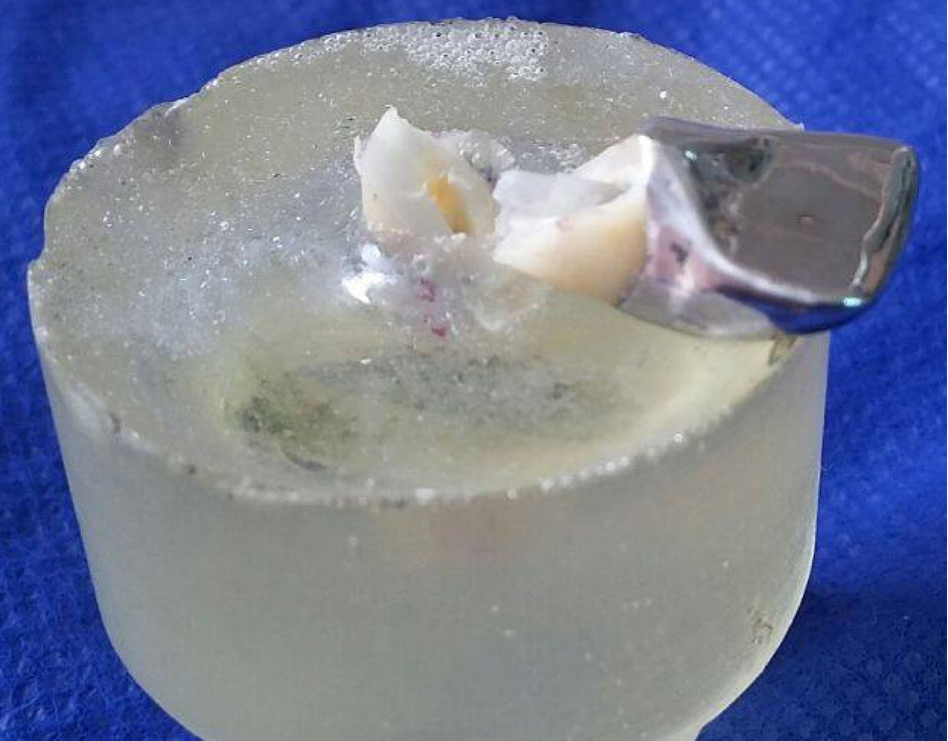
Photograph representing an example of a non-favorable type of failure with an oblique fracture below the cervical third of root that would necessitate extraction of the remaining tooth structure.

The highest overall fracture resistance (N) was observed in control group (827±39.1), followed by CP group [793.8±55.6) N] while the RC group showed the least fracture resistance values [(607.7±54.8) N]. The CR reinforced group showed comparable fracture resistance values to the AP group where the mean values [(727.4±64.4) N] and [(686.2±52.5) N] respectively, while the CP showed comparable fracture resistance to CM group [(771±71.3) N]. The results revealed a significant difference (*P*<0.05) in fracture resistance values of the CP group compared to the CR, AP and RC groups. No significant differences were found among fracture resistance values of the AP group compared to CR groups. Additional analysis with the Tukey test showed significant differences (*P*<0.001) among study groups as well. Predominately restorable failure observed in the C group was 80% while the CP and CR groups recorded 70% as shown in ***Table 3***. However, groups RC, AP and CM recorded a lesser percentage of favorable failure (60%, 40% and 30%, respectively (***Fig. 4***–***5***).

## Discussion

Restoration of root-canal treated teeth with wide root canals using post and core systems still a challenging process as the mismatch between the wide diameter of the flared root canals and standardized rounded diameter of prefabricated post results in thick cement layer. Furthermore, the restoration of these flared root canals may be a compromised treatment as the remaining tooth structure is too weak to tolerate the normal masticatory forces and as a consequence the teeth are liable to fractures^[21^–^22]^. The findings of this *in vitro* investigation support the rejection of the null hypothesis. There was a significant difference in resistance to fracture of endodontically treated teeth with non-flared root canals compared to teeth with flared root canals restored with fiber post, cast post and core and CAM/CAM post and core.

The higher resistance to fracture values recorded for the control group can be explained by the greater amount of remaining dentin. Dentin exhibits considerable plastic deformation that enables resistance of different angles and degrees of load applied during normal occlusal function. Tooth fracture occurs when the applied loads exceeded the tensile strength or proportional limit of dentin as capability of dentin for plastic deformation is decreased. This result is supported by the finding of Zogheib *et al.*^[17]^, who stated that the fracture resistance of endodontically treated teeth is directly proportional to the amount of remaining root canal dentin.

The CP group showed higher resistance to fracture values compared to CR, AP and RC groups. This finding could be related to the modulus of elasticity of VITA Enamic which is comparable to those of the hard tooth structure^[31^–^33]^. Additionally, the difference in chemistry between the composite core material and the epoxy resin of the fiber post matrix, a weak link is formed which is partially responsible for the high failure rate happened in preformed fiber post and core systems. In order to overcome the failure at interfaces, the post and the core should be fabricated as one piece.

The finding of this study showed that the root resistance to fracture of the CR group was significantly higher than the RC group. This finding was supported by a study conducted by Amin *et al.*^[21]^ who reported that reinforcement of the flared post space using composite resin improved the fracture resistance of the weakened roots compared to rehabilitated using luting cement. Additionally, thick layers of resin cement incorporate more bubbles, cracks, or gaps compared to the thin one. These defects initiate a stress concentration zone, which acts as crack raisers and reduces the bond strength of the fiber post to root canal dentin^[25]^. Root fracture might be induced due to the wedging effect of a loose post within a root canal as a result of adhesion failure between root canal dentin and resin cements. The stress accumulated in the post is transferred by the dentin to the outer surface of the tooth. In case of a thin dentinal wall and /or thick resin cement layer, the load required to initiate tooth fracture is minimized, as occurred in groups RC and AP in this study.

Although the use of anatomical post increases the post adaptation to root canal walls and decreases the thickness of resin cement^[22^–^23^,^27]^. Gomes *et al.*^[24]^ showed that the use of composite resin as a root reinforcement material improves fracture resistance of weekend root compared to the use of direct anatomical post. Other studies^[18^,^25^–^26]^ reported that, to strengthen root-canal treated teeth with flared root canals, a firm bond between restorative materials and the root dentin is critical. However, various complicating factors could interfere with firm adhesion. Macedo *et al.*^[22]^ reported that the use of relined post results in increased pull-out bond strengths compared with non-relined post which could be related to the higher adaptation of the post-to-root canal which increases the sustained pressure during cementation process and consequently reduces blister formation in the cement that acts as a stress raiser during testing. Restoration of root-canal treated teeth should be managed with one-piece post and core system with an adequate fit of the post into the root canal to reduce the liability of root fracture.

Failure mode analysis of the test groups revealed the prevalence of a favorable type of failure within C, CP and CR groups. This could be related to the thicker dentin layer in the control group, which increases root fracture resistance. In the other hand, the favorable type of failure observed in the CP group could be a result of its modulus of elasticity that is similar to those of dentin. On the contrary, AP and RC groups showed the prevalence of catastrophic failure. These findings are in accordance with the results of other studies^[22^,^24]^. As the use of glass fiber posts are increasing for restoring endodontically treated teeth, further studies should be conducted to assess not only the appropriate method to restore flared root canal but also the appropriate post length that recommended in such situations.

The present study was an *in vitro* investigation that could not fully replicate the oral conditions which was considered a limitation of this study. Additionally, the specimens were tested under a static loading condition, using three types of post and core systems, and the effect of ferrule was not tested.

In conclusions, the resistance to fracture of wide root canals can be enhanced by using one-piece CAM/CAM post and core as an alternative to the use of either glass fiber post relined with composite resin, increasing the thickness of luting cement or the use of cast post and core system. However, this was an *in vitro* investigation and further *in vivo* studies are necessary.
